# Patient-reported outcomes of ductoscopy procedures for pathologic nipple discharge

**DOI:** 10.1007/s12282-020-01184-y

**Published:** 2020-11-12

**Authors:** M. D. Filipe, J. M. Simons, L. Moeliker, L. Waaijer, M. R. Vriens, P. J. van Diest, A. J. Witkamp

**Affiliations:** 1 Department of Surgery, Cancer Centre, University Medical Centre Utrecht, PO Box 85500, 3508 GA Utrecht, The Netherlands; 2 Department of Pathology, University Medical Centre Utrecht, Utrecht, The Netherlands

**Keywords:** Quality of life, Ductoscopy, Pathologic nipple dischage

## Abstract

**Background:**

Pathologic nipple discharge (PND) is a common complaint often associated with breast cancer. However, when ultrasound and mammography are negative, the chances of malignancy are lower than 5%. Currently, major duct excision and microdochectomy are often recommended to alleviate symptoms and definitely rule out malignancy, but can cause infections and breastfeeding problems. Ductoscopy is a minimally invasive endoscopy technique that allows visualization of the mammary ducts and may not only obviate surgery but also detect malignancy. The aim of this study was to determine quality of life (QOL) after ductoscopy in patients with PND.

**Materials and methods:**

All PND patients referred for ductoscopy between 2014 and 2015 to our hospital were included. Ductoscopy procedures were performed under local anaesthesia in the outpatient clinic. Patients were asked to fill out questionnaires (Breast-Q, EQ-5D-5L and SF-36) on the day of ductoscopy, and after 2 weeks, 3 and 6 months. Additionally, we performed reliability analysis to determine if these questionnaires were suitable for PND patients.

**Results:**

Fifty consecutive patients underwent ductoscopy of whom 47 patients participated in this study. One domain of SF-36 (vitality) varied significantly over time. Breast-Q, SF-36 and EQ-5D-5L showed that QOL after ductoscopy for PND was unaffected by ductoscopy. Success of the ductoscopy procedure was a significant predictor for satisfaction with the result domain.

**Conclusion:**

Ductoscopy is a minimally invasive technique that does not seem to impact QoL of PND patients over time. Breast-Q, SF-36 and EQ-5D-5L seem to be suitable existing QOL tests for PND patients undergoing ductoscopy, whereas SF-36 would require modifications.

## Introduction

Pathologic nipple discharge (PND) is the third most common breast-related complaint, after pain and palpable lumps [1]. PND is defined as unilateral, spontaneous and bloody or serous discharge, usually arising from a single duct orifice of the nipple. PND is regarded as a possible sign of breast cancer and it accounts for 3–5% of surgical breast clinic referrals [2–5]. Ultrasound, mammography and magnetic resonance imaging (MRI) often fail to reveal the underlying cause of PND [6, 7]. Therefore, most women suffering from PND undergo invasive surgical procedures, such as microdochectomy or major duct excision, to rule out malignancy and relieve complaints [6–8]. These surgical procedures are performed under general anaesthesia and are associated with scarring, which may result in breastfeeding difficulties in fertile women [8]. Furthermore, malignancy is found in only 5–8% of these patients [5, 9, 10]. This means around 90–95% of PND patients undergo surgery for benign causes.

Ductoscopy is a minimally invasive micro-endoscopic technique that may obviate the need for invasive surgery in patients with PND. It enables real-time visualization of the milk ducts of the breast. The procedure can be performed under local anaesthesia at the outpatient clinic and is currently used as a diagnostic tool in the work-up of women suffering from PND without suspicious radiological findings [11–18]. Previous studies showed the success of ductoscopy in finding the intraductal lesion causing PND before or during duct excision [19–23]. Moreover, it is possible to remove or laser-destruct intraductal lesions causing PND, thereby preventing surgery [18, 24–26], and new approaches such as auto-fluorescence ductoscopy may improve the detection of precancerous lesions in patients with high risk of developing cancer [27–29]. Despite the increasing relevance of ductoscopy for diagnostic and therapeutic purposes, there are no data available on the impact on the quality of life after ductoscopy. Therefore we evaluated short- and mid-term impact of ductoscopy on quality of life (QOL) of PND patients.

## Materials and methods

### Patient selection

This prospective consecutive study included women who presented with unilateral PND between 2014 and 2015 in the University Medical Centre Utrecht (UMCU) in The Netherlands. Part of these patients were reported before [26]. Inclusion criterion was patients with PND lasting more than at least 3 months. Exclusion criteria for analysis were radiological and/or pathological suspicion of malignancy.

Before ductoscopy, patients underwent imaging consisting of ultrasound and/or mammography. Patients received an additional MRI and/or core needle biopsy (CNB) prior to ductoscopy when there was a palpable mass and/or when ultrasound and/or mammography were BIRADS IV. When these additional tests were negative for malignancy (thereby downgrading the initial BIRADS classification), these patients were eligible for ductoscopy. Either the UMCU (as tertiary referral hospital) or the referring hospital performed the diagnostic work-up. The Medical Research Ethics Committee (METC) of the UMC Utrecht approved the study.

Patients were asked to fill out the Breast-Q, SF-36 and the EQ-5D-5L QOL questionnaires at baseline (T0, day of the ductoscopy), and at 2 weeks (T1), 3 (T2) and 6 months (T3) after ductoscopy. Written informed consent was obtained for all time points.

### Quality of life assessment questionnaires

#### Breast-Q

BREAST-Q is a validated and widely used patient-reported outcome QOL instrument that measures patient satisfaction of the breast after surgical procedures. Categories analysed were sexual and psychological well-being, and physical well-being of the chest. Each scale is summarized in a score ranging from 0 (lowest satisfaction) till 100 (highest satisfaction) [30].

#### SF-36

The Short Form (SF-36) Health Survey is a 36-item, patient-reported survey of general patient health with the following subscales: physical functioning, physical role, bodily pain, social functioning, mental health, emotional status, vitality, and general health perceptions. Each subscale includes 2–10 items and has a score ranging from 0 to 100. Additionally, there are also two large overall subscales for mental health and physical health. Low scores indicate impaired QOL or impaired quality of a certain subscale. SF-36 has been translated into many languages (including Dutch) and validated per language [31, 32].

#### EQ-5D-5L

EQ-5D-5L is a standardized instrument for measuring general health status that consists of descriptive part and a single summary index score. The descriptive part contains five items: mobility, self-care, pain, usual activities and psychological aspects of health. Each of these five items can be scored at five levels (1 = no problems, 2 = slight problems, 3 = moderate problems, 4 = severe problems, 5 = extreme problems). These results are weighted into yield a single summary index score ranging from 0 (worst health) to 1 (best health). The second part of the EQ-5D-5L is the EQ visual analogue scale (VAS) consisting of a 20-cm vertical scale, with a range of 0 (worst health perceived) to 100 points (best health perceived) [33].

### Validation of questionnaires of patients undergoing ductoscopy

Since none of the above questionnaires had been validated for PND patients, we performed reliability analysis by measuring the standardised alpha of each of the domains of the questionnaires. Next, we determined which of the items of the various domains were specifically useful for PND patients. Furthermore, a split-half reliability analysis was performed to further determine internal reliability of the questionnaires. The fact that we asked patients to fill out questionnaires at four different time points allowed a test–retest analysis to determine external reliability of the questionnaires in PND patients.

### Statistics

Normality was determined using Kurtosis, in which *z* values between − 3 and 3 were considered as normally distributed data. Normally distributed continuous data were described by means and standard deviations (SD). In non-continuous not normally distributed data, median and interquartile range were used to describe the data. *P* values below 0.05 were considered to be significant.

In the longitudinal analysis, General Linear Mixed Models was used to determine and compare quality of life over time correcting for possible confounding factors such as age, PND symptoms, final diagnosis made by ductoscopy and/or surgery (benign or malignant) and whether the ductoscopy succeeded or not. Split half correlation analysis and calculation of Cochran’s alpha were performed in order to assess internal reliability. Test–retest using Pearson’s correlation analysis was performed to determine external validity by comparing the test results on the different follow-up periods. Statistical analysis of the database was performed using RStudio 1.2.5001 (with R version: × 64 3.6.2). Statistical packages used were *nmle, tydir, HRQoL, lbscorer* and *psych*.

## Results

Between 2014 and 2015, 50 patients with PND underwent ductoscopy and agreed to fill out questionnaires. Three of these ended up not filling out any of the questionnaires leaving 47 patients for analysis. Table 1 shows the baseline characteristics. The mean age was 51.7 (ranging from 27 to 83). Ductoscopy was successful in 72.3% of cases.

**Table 1:** Baseline characteristics of 47 patients with pathologic nipple discharge studied for quality of life after ductoscopy

	*N* = 47 patients
Age years (SD)	
Affected breast, *N* (%)	51.7 (11.9)
Left	19 (40.4%)
Right	24 (51.1%
Both	4 (8.5%)
Successful ductoscopy, *N* (%)	
Yes	34 (72.3%)
No	13 (27.7%)
Diagnosis of ductoscopy, *N* (%)	
Normal	10 (21.3%)
Papilloma	11 (23.4%)
Epithelial lesions	3 (6.4%)
Suspicious for malignancy	5 (10.6%)
Other	5 (10.6%)
NA	13 (27.7%)
Ultrasound BI-RADS classification, *N* (%)	
BI-RADS I	18 (38.3%)
BI-RADS II	20 (42.6%)
BI-RADS III	1 (2.1%)
Not performed	8 (17.0%)
Mammography BI-RADS classification, *N* (%)	
BI-RADS I	33 (70.2%)
BI-RADS II	9 (19.1%)
BI-RADS III	2 (4.3%)
BI-RADS IV	1 (2.1%)
Not performed	2 (4.3%)
MRI BI-RADS classification, *N* (%)	
BI-RADS I	7 (14.7%)
BI-RADS II	5 (10.6%)
BI-RADS III	2 (4.3%)
BI-RADS IV	1 (2.1%)
Not performed	32 (68.1%)
Diagnosis after possible surgery, *N* (%)	
Benign	6 (12.8%)
Malignant	4 (8.5%)
Not performed	37 (78.7%)
PND stopped after (attempted) ductoscopy, *N* (%)	
Yes	18 (38.3%)
No	29 (61.7%)

*SD* standard deviation, *BI-RADS* Breast Imaging Reporting and Data System, *MRI* magnetic resonance imaging, *NA* not applicable

### Breast-Q

Table 2 shows the average scores per Breast-Q domain over the follow-up period. Average psychosocial well-being was 69.7 out of 100 at baseline and 66.4, 70.3 and 69.8 at 2 weeks, 3 and 6 months after ductoscopy, respectively. Mean physical well-being of the chest was 73.8 out of 100 at baseline, 73.0 after 2 weeks, 78.7 after three and 78.9 after 6 months. Patients scored 29.6 out of 100 for sexual well-being on the day of ductoscopy, 30.4 after 2 weeks, 20.1 after three and 22.8 after 6 months. Satisfaction with the result was scored on average 53.0 points 2 weeks after ductoscopy, 50.0 after 3 and 47.0 after 6 months. None of the domains showed statistically significant differences over time after correction for age, whether or not PND prevailed, diagnosis after possible surgery and success of ductoscopy. However, success of ductoscopy was a significant predictor of satisfaction with the result domain.

**Table 2:** Results of 3 questionnaires (Breast-Q, EQ-5D-5L, SF-36) to assess quality of life over time of patients with pathologic nipple discharged undergoing ductoscopy

	T0	T1	*P* value	T2	*P* value	T3	*P* value
	Mean (SD)	Mean (SD)		Mean (SD)		Mean (SD)	
Breast-Q							
A	69.7 (16.17)	66.4 (16.3)	0.132	70.35 (20.67)	0.689	69.76 (17.04)	0.971
B	73.79 (15.08)	73 (18.01)	0.145	78.7 (18.73)	0.378	78.88 (12.94)	0.478
C	29.57 (13.63)	30.36 (16.69)	0.149	20.14 (15.11)	0.157	22.75 (15.46)	0.300
D	NA	53.03 (24.3)	NA	50.04 (20.23)	0.432	47.00 (27.98)	0.497
EQ-5D-5L							
EQ-5D (*100)	86.24 (14.49)	86.53 (13.9)	0.875	84.89 (17.72)	0.447	90.79 (10.29)	0.381
VAS	81.43 (11.54)	79.2 (12.13)	0.256	76.57 (13.62)	0.075	80.71 (13.08)	0.378
SF-36							
PF	92.50 (8.11)	92.8 (7.92)	0.700	87.95 (18.5)	0.305	91.18 (11.93)	0.444
RP	71.43 (38.32)	72 (40.39)	0.919	75 (43.3)	0.604	75 (41.46)	0.184
BP	73.32 (19.07)	74.36 (22.1)	0.891	80.74 (20.36)	0.089	81.53 (17.5)	0.218
GH	72.94 (18.69)	71.2 (19.05)	0.818	68 (21.31)	0.296	70.18 (21.1)	0.320
VT	*66.25 (17.83)*	*58 (17.74)*	*0.001*	*60.87 (19.81)*	*0.014*	*62.19 (17.41)*	*0.048*
SF	86.61 (16.29)	84 (20.58)	0.063	86.41 (23.21)	0.323	89.71 (14.14)	0.523
RE	80.95 (36.77)	77.33 (36.92)	0.299	74.6 (43.34)	0.506	80.39 (35.47)	0.655
MH	75.14 (13.96)	73 (20.23)	0.399	71.48 (20.76)	0.273	75.75 (15.63)	0.970

Italic values are statistically significant

*Breast-Q* Breast questionnaire, *EQ-5D-5L* European Quality of Life, *SF-36* Short form health survey, *VAS* Visual analogue score, *PF* Physical functioning, *RP* role physical, *BP* bodily pain, *GH* general health, *VT* vitality, SF = Social functioning, RE = Role emotional, MH = Mental health, *PCS* [overall] physical health, *MCS* [overall] mental health, *A* psychosocial well-being, *B* physical well-being: chest, *C* sexual well-being, *D* satisfaction with outcome, *T0* Day of ductoscopy, *T1* two weeks after ductoscopy, *T2* three months after ductoscopy, *T3* six months after ductoscopy, *SD* standard deviation, *NA* not applicable, cursive = significant differences and *p* value = differences compared to survey at T0 and are corrected for age, PND symptoms after ductoscopy, whether or not operated and diagnosis in operated patients

### EQ-5D-5L

Table 2 displays mean scores for the EQ-5D-5L questionnaires. Average descriptive score was 86.2 out of 100 in patients at baseline, 86.5, 2 weeks after ductoscopy, 84.9 after three and 90.8 after 6 months. Mean VAS score was 81.4 on the day of the ductoscopy, 79.2, 2 weeks later, 76.6 three and 80.7, six months after ductoscopy. There were no statistically significant differences in scores during follow-up. Age, PND, possible surgery, diagnosis of possible surgery and success of ductoscopy were not confounders for the EQ-5D-5L.

### SF-36

Table 2 shows the average sum scores of the different domains of the SF-36 questionnaires. Physical functioning, role physical scores, bodily pain, general health, social functioning, role emotional scores and mental health showed no statistical differences in scores during follow-up. Vitality scores dropped statistically significantly from 66.2 at baseline to 58.0 after 2 weeks, 60.9, three months and 62.2 after 6 months. Age was only a significant confounder for the physical functioning domain. Success of ductoscopy, diagnosis after possible operation, whether or not PND stopped and whether or not a patient was operated did not statistical significant influence on any of the SF-36 domains.

### Internal and external reliability analysis

Table 3 shows the results of the internal and external reliability analysis. For BREAST-Q, reliability for psychosocial and physical well-being did not increase if individual items were dropped. Test–retest correlations of psychosocial, physical and sexual well-being and satisfaction of outcome were 0.68, 0.79, 0.67 and 0.63, respectively. Two items (“Comfortable/at ease during sexual activity” and “Confident sexually”) correlated negatively with sexual well-being domain and if the former would be dropped, standardised alpha would increase. If the item “I would encourage other women in my situation to have breast reconstruction surgery” would be dropped, the reliability of satisfaction of outcome would increase.

**Table 3:** Internal and external validity of three quality of life questionnaires in patients with PND undergoing ductoscopy

	Std. alpha	Split half reliability	Test–retest reliability
Breast-Q			
A	0.92	0.80	0.68
B	0.93	0.68	0.79
C	0.65	0.52	0.67
D	0.86	0.78	0.63
EQ-5D-5L			
EQ-5D	0.51	0.46	0.75
VAS	NA	NA	0.78
SF-36			
PF	0.86	0.58	0.70
RP	0.87	0.74	0.92
BP	0.76	0.61	0.72
GH	0.82	0.21	0.76
VT	0.66	− 0.41	0.79
SF	0.82	− 0.69	0.85
RE	0.90	0.77	0.55
MH	0.85	− 0.41	0.87

*Breast-Q *Breast questionnaire, *EQ-5D-5L* European Quality of Life, *SF-36* Short form health survey, *VAS* Visual analogue score, *PF* physical functioning, *RP* role physical, *BP* bodily pain, *GH* general health, *VT* vitality, *SF* social functioning, *RE* role emotional, *MH* mental health, *PCS* = [overall] physical health, *MCS* [overall] mental health, *A* psychosocial well-being, *B* physical Well-being: chest, *C* sexual well-being, *D* satisfaction with outcome, *NA* not applicable

For EQ-5D-5L, the item “mobility” was not taken into the analysis because there was no variance. If the “anxiety and depression” item would be dropped, the standardised alpha would increase. Split test–retest analysis determining external validity showed a correlation of 0.75 for the descriptive part and 0.78 on the VAS part.

For SF-36, dropping individual physical functioning items would not increase reliability. If the first item of role physical (“Cut down the amount of time you spent on work or other activities”) would be dropped, the standardised alpha would increase. If the first item of role emotional (“Cut down the amount of time you spent on work or other activities”) would be dropped, the standardised alpha would increase. Dropping individual items of general health would not increase internal validity. However, the second (“I seem to get sick a little easier than other people”) and fourth (“I expect my health to get worse”) items showed strong negative correlations. Dropping individual items would not improve reliability of the social functioning domain. The first item of social functioning (“During the past 4 weeks, to what extent has your physical health or emotional problems interfered with your normal social activities with family, friends, neighbours, or groups”) correlated strongly negative with the domain. For bodily pain, dropping an individual item would not improve reliability. For vitality, the two first items (“Did you feel full of pep?” and “Did you have a lot of energy?”) were strongly negative correlated and if the former was dropped, the domain would be more reliable. For mental health, dropping the first item (“Have you been a very nervous person?”) would increase reliability while other items (“Have you felt calm and peaceful?” and “Have you been a happy person?”) negatively correlated to the mental health score. Test–retest analysis showed a correlation of 0.87 for external validity.

## Discussion

In this study, we evaluated QOL of patients with PND that underwent ductoscopy. No statistically significant changes were observed in the overall scores of the Breast-Q, SF-36 and EQ-5D-5L questionnaires at baseline compared to 2 weeks, 3 and 6 months after ductoscopy, indicating that ductoscopy does not negatively influence QOL. These findings further support the use of ductoscopy, which is performed more and more for both diagnostic and therapeutic purposes [26, 34, 35]. Additionally, this study showed that the all domains of Breast-Q, and EQ-5D-5L were stable over time and are thereby useful questionnaires for PND patients, while SF-36 was as a whole less useful and would require modifications.

For Breast-Q the average patient was satisfied with the outcome, with scores higher than reported by studies that assessed QOL of patients undergoing breast conserving surgery [36, 37], and all domains scored well in internal and external validity. Regarding average sexual well-being, this was reported to be stably low in our study, likely associated with PND itself. For EQ-5D-5L, both descriptive scores and VAS scores were stable over time. For SF-36, the vitality domain showed low reliability scores, and omitting this domain would make this questionnaire more useful for PND patients. For all questionnaires, scores for ductoscopy were overall similar to or higher than breast conserving therapy [38–41], favouring ductoscopy as a procedure. To the best of our knowledge, there are no studies describing QOL in PND patients undergoing surgery.

Ductoscopy is often used to detect the cause underlying PND, which in the majority of cases is a benign lesion [23, 35, 42–45]. A meta-analysis showed that ductoscopy has a sensitivity of 94% in detecting intraductal lesions of any type, but cannot reliably discriminate between benign and malignant lesions [46]. Another network meta-analysis showed that ductoscopy has a significantly higher diagnostic accuracy than MRI for the detection of malignancy in patients with PND and negative ultrasound and/or mammography [47]. Therefore, ductoscopy is more useful when conventional imaging is negative [26]. New techniques in ductoscopy are currently being developed and explored to improve the sensitivity for the detection of (pre)malignant lesions. One of these examples is combining ductoscopy with auto-fluorescence imaging [27–29]. This addition to ductoscopy may help to detect precancerous lesions in high-risk breast cancer groups since auto-fluorescence has shown to be effective for the detection of precancerous lesions of epithelial origin (such as oesophageal, lung, bronchial and colon cancers) [48–52]. Additionally, the development of therapeutic options such as intraductal laser ablation further increase the utility of ductoscopy [53]. This means that there is an increasing potential for ductoscopy not only for diagnostic but also for therapeutic purposes. Invasive surgery can be avoided in the majority of patients with PND that undergo ductoscopy as the initial procedure which is a major advantage [26].

Limitations of this study are our relatively small study population and the relatively short follow-up period. Nevertheless, this is the first study to report on QOL following ductoscopy and we do not expect any significant changes in QOL beyond 6 months, since ductoscopy is a minimally invasive procedure.

To conclude, ductoscopy is a minimally invasive endoscopic technique that is currently used to detect, and sometimes treat, intraductal lesions causing PND. This QOL study further supports the use of ductoscopy, as it shows no negative influence on short or mid-term QOL in PND patients. Breast-Q and EQ-5D-5L appear to be useful existing questionnaires for the assessment of QOL in PND patients, while SF-36 would require modifications. Nevertheless, development of a questionnaire specific to PND patients may even be better.
